# Knowledge, attitude, and practice of nurses towards frailty syndrome: a multicenter cross-sectional survey in Fujian Province, China

**DOI:** 10.3389/fmed.2026.1659941

**Published:** 2026-02-11

**Authors:** Hong Chen, Maohe Chen, Ruina Luan, Chunying Chen, Meiyun Su, Pingping Hong, Jun Guo, Bifang Zhu, Hanbin Lin, Ping Lin

**Affiliations:** 1 Geriatrics Department (Including Rheumatology and Immunology), The First Hospital of Putian City, Putian, Fujian, China; 2 Department of Respiratory and Critical Care Medicine, Affiliated Hospital of Putian University, Putian, Fujian, China; 3 Department of Nursing, The First Hospital of Putian City, Putian, Fujian, China; 4 Department of Ophthalmology, The First Hospital of Putian City, Putian, Fujian, China; 5 Department of Neurology, Putian City Xiuyu District Hospital, Putian, Fujian, China; 6 Comprehensive Ward, Nanri Branch of Putian First Hospital (Nanri Town Health Center), Putian, Fujian, China; 7 Department of Basic Public Health, Community Health Service Center of Fenghuangshan Street, Putian, Fujian, China

**Keywords:** attitude, cross-sectional study, frailty, knowledge-practice gap, nursing care

## Abstract

**Background:**

Frailty is a global geriatric challenge requiring integrated nursing management. However, the mechanism driving nurses’ practice behaviors remains unclear. This study aimed to evaluate the status of knowledge, attitude, and practice (KAP) regarding frailty syndrome among nurses and identify the independent predictors of clinical practice

**Methods:**

A multicenter cross-sectional survey was conducted among 598 registered nurses in Fujian, China, using a structured questionnaire. Data were analyzed using descriptive statistics and multiple linear regression to identify predictors of practice scores while adjusting for potential confounders

**Results:**

Participants demonstrated foundational theoretical knowledge of frailty definitions but exhibited substantial gaps in practical application, particularly in identifying risk factors and conducting comprehensive assessments. While univariate analysis suggested associations between practice scores and factors like knowledge, age, and education, the multivariate model revealed that Attitude was the sole robust independent predictor (β = 0.67, *p* < 0.001). Notably, knowledge and demographic variables lost statistical significance after adjustment (*p* > 0.05), indicating a distinct “knowledge-practice gap” where theoretical competence does not automatically translate into clinical action without intrinsic motivation

**Conclusion:**

A significant dissociation exists between nurses’ theoretical knowledge and their actual practice behaviors in frailty care. Since attitude acts as the decisive mediator overriding demographic barriers, future clinical pathways should shift from purely didactic training to fostering a proactive professional culture. Interventions must prioritize motivational strategies to bridge the knowledge-practice gap and optimize patient safety outcomes.

## Introduction

Frailty syndrome (FS) is a multidimensional geriatric condition characterized by a decline in physiological reserve and an increased vulnerability to stressors, leading to higher risks of falls, hospitalization, and death (1–3). As global populations age, FS has become a critical public health challenge. In China, where the older adult population exceeds 264 million, approximately 10% are affected by FS (4). This demographic shift necessitates a healthcare approach that goes beyond traditional disease management to embrace the “Quadruple Aim” (5): improving population health, reducing costs, enhancing the patient experience, and improving the work-life of healthcare providers.

Effective management of frailty is not merely a clinical task but a fundamental strategy for ensuring Patient Safety. Missed screening for frailty risk factors often leads to preventable adverse events, such as falls and adverse events related to drug-to-drug interactions, particularly in older patients with multimorbidity and polypharmacy. Furthermore, identification of biopsychosocial risk factors—including nutritional status, cognitive decline, psychological well-being, and social isolation—is critical for early intervention. Therefore, establishing Innovative Diagnostic and Therapeutic Pathways (6, 7) is essential to address the fragmentation among professionals, enabling the early identification of Frailty Syndrome and the integrated management of the multifactorial conditions that can lead to adverse events. However, the sustainability of such programs requires continuous “renovation.” Just as clinical networks must adapt to improve efficiency, frailty care programs must evolve by integrating multidisciplinary collaboration and advanced screening tools like the Comprehensive Geriatric Assessment (CGA) (8).

Nurses can contribute significantly to the early detection of frailty risk factors, promoting prevention and health promotion interventions in pre-frail older adults. Through the use of multidimensional tools that require less intensive geriatric expertise, nurses are uniquely positioned to identify risk factors for biopsychosocial frailty and educate older adults on adopting a healthy lifestyle. However, the successful implementation of standardized frailty pathways relies heavily on the competence of the nursing workforce. Despite the growing emphasis on geriatric care in China, data regarding nurses’ specific Knowledge, Attitudes, and Practices (KAP) toward FS remain limited.

Understanding the current KAP status is the prerequisite for designing effective educational interventions and renovating clinical workflows. Therefore, this study aims to: (1) evaluate the current level of knowledge, attitude, and practice regarding FS among nurses in Fujian, China; (2) identify the gap between theoretical knowledge and clinical execution; and (3) determine the demographic and professional factors influencing frailty care behaviors. The findings will provide an evidence base for developing targeted training programs to achieve the Quadruple Aim in geriatric nursing.

## Materials and methods

This study was conducted and reported in accordance with the Strengthening the Reporting of Observational Studies in Epidemiology (STROBE) (9) statement for cross-sectional studies. The completed STROBE checklist is provided as Supplementary material 1.

### Participants and setting

A cross-sectional, multicenter survey was conducted to assess the knowledge, attitudes, and practices (KAP) of nurses regarding frailty syndrome in Fujian Province, China. In the Chinese hospital classification system, these institutions are categorized based on their bed capacity and functional role: Tertiary hospitals are large comprehensive medical centers (typically > 500 beds) providing high-level specialized care, medical education, and research; Secondary hospitals are regional facilities (100–499 beds) offering medical services to local districts; and Community hospitals (Primary healthcare institutions) focus on basic public health, prevention, and rehabilitation services. A self-administered structured questionnaire was distributed through the Questionnaire Star software platform via WeChat from June to October 2023. The inclusion criteria for participants were as follows: (a) registered professional nurses; (b) working in the frontline of medical institutions; and (c) having at least 1 year of clinical work experience. Exclusion criteria included (a) nurses currently working in logistics or administrative departments, (b) nurses with less than 1 year of clinical work experience, (c) individual or part-time nurses, and (d) those unwilling to participate in the survey. A total of 625 nurses participated, and after excluding invalid responses, 598 valid questionnaires were obtained, yielding an effective response rate of 95.7%.

The minimum required sample size was estimated using G*Power software (version 3.1), setting a significance level (α) of 0.05, a power (1-β) of 0.80, and a medium effect size (*f* = 0.25) based on previous similar surveys. Considering a potential invalid response rate of 10%, the final target sample size was set at 550 participants.

The study protocol was approved by the Human Research Ethics Committee of The First Hospital of Putian City, and informed consent was obtained from all participants prior to data collection. To reduce potential recall and social desirability biases, all responses were collected anonymously and voluntarily, and participants were assured that their information would remain confidential and be used solely for academic research purposes.

### Questionnaire design

The self-administered questionnaire was developed and evaluated based on the expert consensus and guidelines of frailty syndrome by 3 experts (including one nursing manager, one nursing practitioner, and one geriatrist). The survey including four parts (i) Basic characteristics of participants: gender, age, education, hospital grade, Department, and working experience; (ii) Knowledge part: including 4 mandatory single-choice items and 7 multiple-choice item about definition, judgment criteria, and other basic concept of FS; (iii) Attitude part: concerning and willingness about FS, scale from strongly disagree to strongly agree; (iv) Practice part: self-reported experiences in clinical practice about preventing and patients education of FS, scale from always to never. Knowledge items were scored objectively (1 point for correct, 0 for incorrect), with multiple-choice items awarding points for each correct option identified. Attitude and Practice parts used 4-point Likert scales. Supplementary material 2 provided detailed explanations and numerical score of each question.

### Data analysis

Descriptive statistics were used to summarize demographic variables, presented as frequencies and percentages for categorical variables and means with standard deviations for continuous variables. One-way analysis of variance (ANOVA) was performed to examine the associations between demographic characteristics and both knowledge and attitude scores. Variables with *p*-values < 0.05 in the univariate analysis were subsequently included in multivariate analysis. Multiple linear regression analysis was performed to identify independent predictors of frailty-related nursing practice behaviors. The dependent variable was the total practice score (continuous, range: 7–28). Variables with *p* < 0.05 in the univariate analysis were entered into the multivariate model. To ensure the robustness of the model, collinearity diagnostics were conducted using the Variance Inflation Factor (VIF). All VIF values were found to be less than 5, indicating no severe multicollinearity among independent variables (e.g., age, professional rank, and years of working). Model fit was evaluated using the Coefficient of Determination (R^2^) and the ANOVA F-test. All statistical analyses were performed using SPSS version 22.0 (IBM, Chicago, IL, United States), and a *p* < 0.05 was considered statistically significant.

## Results

### Characteristics of participants

**FIGURE 1:**
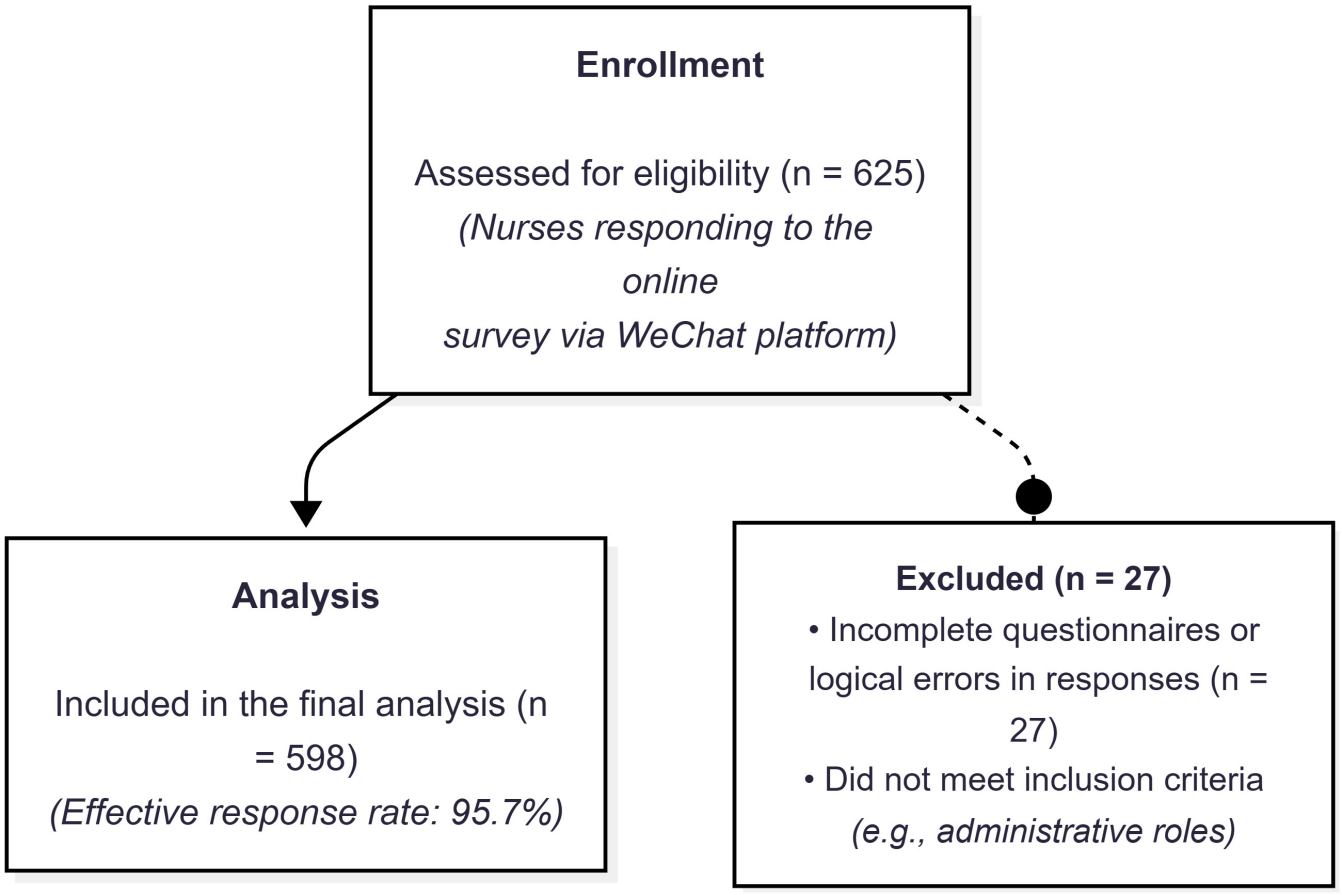
Flow diagram of participant recruitment and selection process.

The participant recruitment and selection process is illustrated in Figure 1. A total of 625 nurses initially responded to the online survey. After excluding 27 questionnaires due to incompleteness or failure to meet inclusion criteria (e.g., administrative roles or logical errors), 598 valid responses were included in the final analysis, yielding an effective response rate of 95.7%. The demographic characteristics of these participants are outlined in Table 1. Among these participants, 550 nurses (97.52%) identified as female. Additionally, 203 nurses (35.99%) fell within the age range of 18-29, while 248 nurses (43.97%) were aged between 30 and 39. In terms of hospital affiliation, 228 nurses (40.43%) were employed in tertiary hospitals, while 233 nurses (41.31%) worked in secondary hospitals. The majority of nurses possessed a college degree (81.7%), with only 97 nurses having attended a technical secondary school and a mere 6 nurses holding a master’s degree or higher. Furthermore, 525 nurses occupied junior or intermediate positions within their respective institutions. Approximately 50% of the participants (249) possess a professional background exceeding a decade in the workforce, while the participants were drawn from diverse departments.

**TABLE 1:** Characteristics of nurses.

Variables	N	Percentage (%)
Respondents	564	–
**Gender**		
Male	14	2.48
Female	550	97.52
**Age**		
18–29	203	35.99
30–39	248	43.97
>40	113	20.04
**Type of medical institution**		
Tertiary hospitals	228	40.43
Secondary hospitals	233	41.31
Community hospitals	103	18.26
**Education**		
Technical secondary school	97	17.20
College degree	461	81.74
Master degree and above	6	1.06
**Professional rank**		
Junior	345	61.17
Intermediate	180	31.91
Senior	39	6.91
**Years of working**		
<5 years	172	30.50
5–10 years	143	25.35
10–15 years	132	23.40
> 15 years	117	20.74
**Departments**		
Internal medicine department	176	31.21
Surgery department	274	48.58
General practice department	35	6.21
Geriatrics department	37	6.56
Emergency department and ICU	42	7.45

ICU, intensive care unit.

### Nurses’ knowledge of frailty syndrome

**FIGURE 2:**
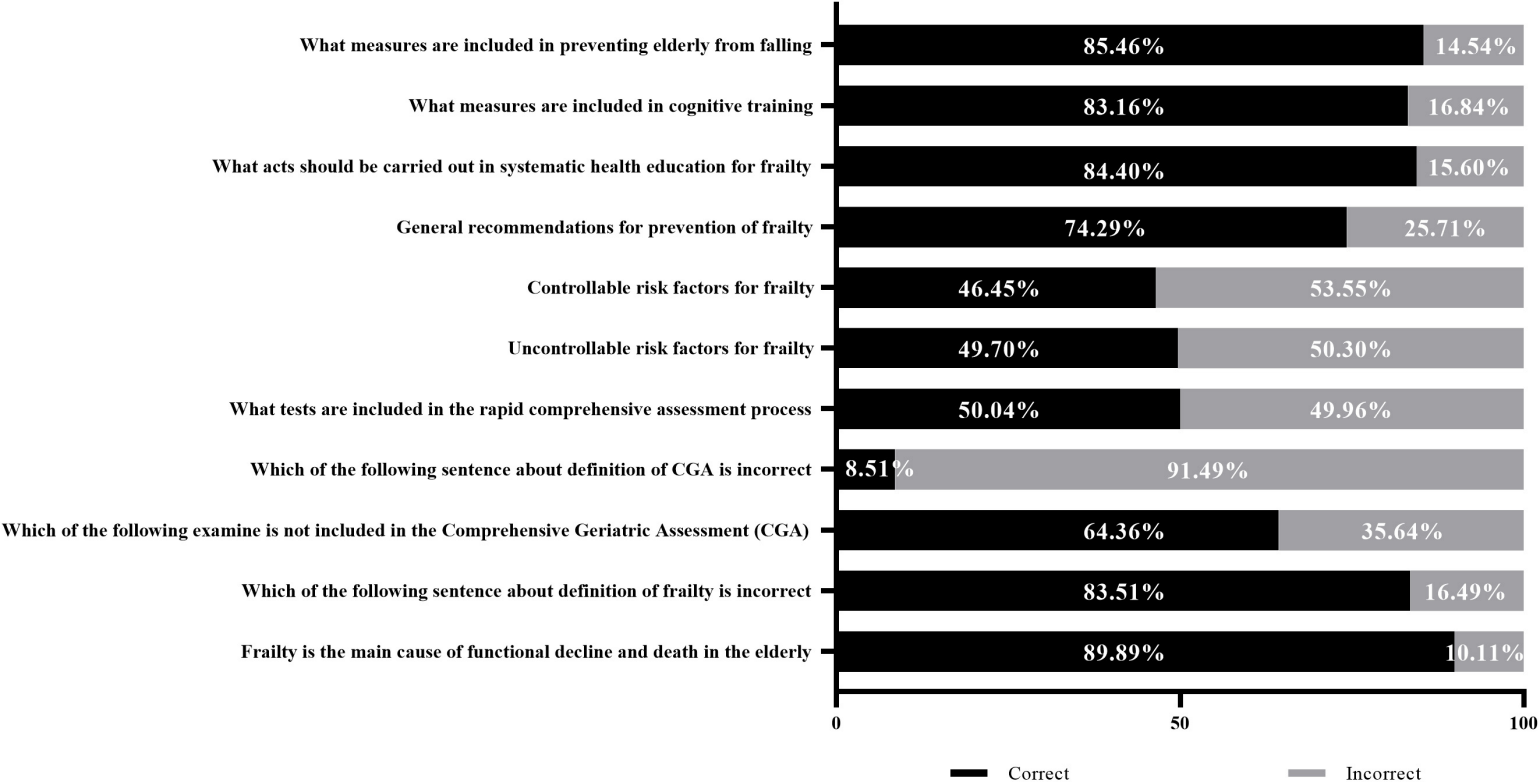
Nurses’ Knowledge regarding frailty syndrome.

According to the findings depicted in Figure 2, the study revealed a distinct dichotomy in nurses’ knowledge structure. While over 80% of participants demonstrated a proficient comprehension of the conceptual definition of frailty syndrome, practical knowledge was significantly lacking. Specifically, only 46.45% and 49.70% of nurses correctly identified controllable and uncontrollable risk factors, respectively. Similarly, a significant proportion of nurses exhibited a deficiency in their understanding of the Comprehensive Geriatric Assessment and other assessments related to frailty syndrome. Furthermore, it is noteworthy that specific misconceptions persisted: 15.60% held misconceptions regarding providing patients with education on preventing frailty; 16.84% showed a lack of knowledge in the context of cognitive training; while 14.54% held misconceptions regarding fall prevention measures for older individuals.

### Nurses’ attitude toward frailty syndrome

The results of the nurses’ attitude toward FS reporting indicate that the majority of nurses’ recognize the importance of nursing care for FS. 95.58% agreed that prevention of frailty is important part of nursing service, 87.11% are willing to voluntary participate in education of frailty, 95.40% believe that nurses should conduct a dynamic assessment frailty for hospitalized patients. Regarding the issue of effect of nursing care towards FS, 25.85% of participants disagree and 4.77% strongly disagree that poor nursing care was critical risk factor for frailty syndrome. The results are shown in Table 2.

**TABLE 2:** Attitudes of nurses toward frailty syndrome.

Items	Strongly agree (%)	Agree (%)	Disagree (%)	Strongly disagree (%)
Do you agree the prevention of Frailty diseases is very important?	33.63	62.65	1.24	2.48
Do you agree the prevention of Frailty important part of nursing service?	28.50	67.08	2.12	2.30
Do you willing to voluntary participate in education of frailty to patients and other people?	19.20	67.91	10.05	2.84
Do you believe it’s nurse should conduct a dynamic assessment of frailty for in hospital patients?	22.83	72.57	3.72	0.88
I support that poor nursing care was critical risk factor for frailty syndrome	22.30	47.08	25.85	4.77

### Nurses’ practice toward frailty syndrome

Regarding the practice of recognize and preventing risk factors for frailty syndrome among nurses. 38.1% of participants always and 30.8% often make risk assessment for falling of patient. Furthermore, 60.8 and 29.7% take action to prevent hospitalized patients from falling. In contrast, nearly half of the participants recognized they rarely or never participate in health education of frailty or sarcopenia. As for the health education of exercise, healthy lifestyle, and mental health, 77.2, 74.0, and 75.8 of the participants always/often provide guidance for their patients. For concomitant diseases such as hypertension, diabetes, and cancer, 38.4 and 39.5% of the participants provide extra care. The results are shown in Table 3.

**TABLE 3:** Nurses’ practice for frailty syndrome.

Items	Always (%)	Often (%)	Rarely (%)	Never (%)
Risk assessment for falling in patients	38.12	30.85	18.44	12.59
Fall risk prevention of hospitalized patients	60.82	29.79	7.98	1.42
Health education of frailty or sarcopenia with patients	22.87	29.26	29.61	18.26
Health education of exercise with older patients	37.06	40.25	20.57	2.13
Health education of healthy lifestyle and nutritional support with older patients	34.04	40.07	22.52	3.37
Guidance on the mental health of older patients	36.35	39.54	21.10	3.01
Extra care for concomitant disease	38.48	39.54	19.68	2.30

### Differences in knowledge and attitude scores across nurse characteristics

The study revealed significant differences in nurses’ knowledge and attitude scores based on their personal characteristics. Specifically, nurses’ age, type of medical institution, education level, professional rank, years of working experience, and department significantly influenced both knowledge and attitude scores (*p* < 0.05). In detail, nurses with a bachelor’s degree, intermediate professional rank, 5–10 years of working experience, and those working in the Geriatrics Department exhibited higher knowledge scores. Nurses in tertiary hospitals also had significantly higher knowledge scores compared to those in non-tertiary hospitals (*p* < 0.001).

Significant differences were also observed between nurses’ characteristics and their attitude scores. Specifically, older nurses, those with higher educational backgrounds, and those with more than 15 years of working experience had lower attitude scores (*p* < 0.05). In contrast, nurses in the Geriatrics Department demonstrated the highest scores in both knowledge and attitude. The differences in nurses’ knowledge and attitude scores are presented in Table 4.

**TABLE 4:** The relationship between nurses’ characteristics and knowledge and attitude scores.

Variable	Knowledge score (0–34)			Attitude score (0–20)		
	Average	SD	*P*	Average	SD	*P*
**Gender**						
Male	26.71	5.48	0.503	17.29	2.84	0.485
Female	26.05	3.63		15.92	2.48	
**Age**						
18–29	26.22	3.81	0.382	16.05	2.27	**0.010**
30–39	26.15	3.55		16.06	2.49	
40–49	25.72	3.69		15.79	2.64	
>50	25.04	3.84		14.65	3.56	
**Type of medical institution**						
Tertiary hospitals	26.43	2.65	**< 0.001**	15.29	2.72	0.233
Secondary hospitals	26.25	3.58		16.08	2.36	
Community hospitals	25.71	4.14		16.14	2.49	
**Education**						
High school and below	25.10	4.31	**0.019**	15.93	2.34	**< 0.001**
College degree	26.30	3.48		16.01	2.39	
Master degree and above	23.50	4.28		12.00	7.21	
**Professional rank**						
Junior	25.98	3.84	**0.005**	15.87	2.39	**0.001**
Intermediate	26.26	3.18		16.16	2.44	
Senior	25.90	4.34		15.79	3.56	
**Years of working**						
<5 years	25.59	4.12	**0.010**	15.92	1.96	**<0.001**
5–10 years	26.71	3.14		16.14	2.46	
10–15 years	26.11	3.55		16.11	2.83	
>15 years	25.93	3.67		15.61	2.82	
**Departments**						
Internal medicine department	26.44	3.56	**< 0.001**	15.97	2.64	**0.001**
Surgery department	25.59	4.02		15.89	2.28	
General practice department	25.97	3.05		15.91	3.62	
Geriatrics department	27.38	2.37		16.49	2.04	
Emergency department and ICU	26.48	2.73		15.90	2.53	

ICU, intensive care unit. Data presentation: Values are expressed as mean ± standard deviation (SD). Hypothesis Testing Framework: The null hypothesis (H_0_) posited no significant difference in mean scores across the subgroups. Statistical tests: *P*-values were derived using Independent Samples *t*-test for variables with two categories (Gender) and One-way Analysis of Variance (ANOVA) for variables with three or more categories (Age, Type of Medical Institution, Education, Professional Rank, Years of Working, Departments). Significance: A *p* < 0.05 indicates a statistically significant difference in mean scores between groups. Bold values indicate statistical significance (*p* < 0.05).

### Predictive factors for nursing practice behaviors

Univariate and multivariate linear regression analyses were performed to identify predictors of nurses’ frailty care practice behaviors (Table 5). In the univariate analysis, several factors appeared to be significantly associated with practice scores. Specifically, higher knowledge scores (β = 0.14, *p* = 0.012) and attitude scores (β = 0.68, *p* < 0.001) were positively correlated with better practice behaviors. Conversely, nurses with advanced age (>50 years), higher educational levels (Master’s degree or above), intermediate professional titles, and longer working experience (>10 years) exhibited significantly lower practice scores. Regarding the type of institution, nurses in secondary hospitals showed higher practice scores compared to those in tertiary hospitals. However, after adjusting for potential confounding factors in the multivariate linear regression model, only Attitude remained a significant independent predictor. The results indicated that for every one-point increase in the attitude score, the practice score increased by 0.67 points (95% CI: 0.51 to 0.82, *p* < 0.001). Notably, Knowledge was no longer a statistically significant predictor in the multivariate model (β = 0.05, *p* = 0.359). Similarly, demographic and professional characteristics—including age, educational level, professional title, years of work experience, and hospital type—lost their statistical significance after adjustment (*p* > 0.05). This suggests that the variations in practice behaviors initially observed across different demographic groups were likely mediated by the nurses’ attitudes rather than being direct effects of the demographic factors themselves.

**TABLE 5:** Univariate and multivariate linear regression analysis of factors associated with nurses’ frailty care practice scores.

Variables	Univariate analysis		Multivariate analysis	
	Coefficient (β) (95% CI)	*P*-value	Coefficient (β) (95% CI)	*P*-value
Knowledge score	0.14 (0.03 to 0.25)	**0.012**	0.05 (-0.06 to 0.16)	0.359
Attitude score	0.68 (0.54 to 0.83)	**< 0.001**	0.67 (0.51 to 0.82)	**< 0.001**
**Professional rank**				
Junior	Ref		Ref	
Intermediate	-1.31 (-2.17 to -0.44)	**0.003**	-0.82 (-1.89 to 0.25)	0.134
Senior	-0.56 (-2.16 to 1.03)	0.486	0.07 (-2.00 to 2.14)	0.946
**Years of working**				
< 5 years	Ref		Ref	
5–10 years	-0.81 (-1.87 to 0.26)	0.137	-0.74 (-1.84 to 0.36)	0.185
10–15 years	-1.85 (-2.94 to -0.77)	**0.001**	-1.29 (-2.67 to 0.09)	0.068
> 15 years	-1.70 (-2.82 to -0.57)	**0.003**	-1.35 (-3.33 to 0.63)	0.18
**Departments**				
Internal medicine	Ref		Ref	
Surgery	-0.66 (-1.57 to 0.26)	0.159	-0.53 (-1.40 to 0.33)	0.227
General practice	-1.96 (-3.71 to -0.21)	**0.028**	-1.37 (-3.04 to 0.31)	0.109
Geriatrics	-0.99 (-2.70 to 0.72)	0.256	-1.37 (-2.98 to 0.24)	0.096
Emergency/ICU	-1.28 (-2.90 to 0.35)	0.123	-1.31 (-2.85 to 0.23)	0.096
**Age (years)**				
18–29	Ref		Ref	
30–39	-1.50 (-2.39 to -0.61)	**0.001**	-0.34 (-1.46 to 0.77)	0.547
40–49	-1.06 (-2.25 to 0.13)	0.082	0.75 (-1.08 to 2.57)	0.421
> 50	-2.48 (-4.54 to -0.41)	**0.019**	0.03 (-2.71 to 2.76)	0.984
**Education**				
High school	Ref		Ref	
College degree	-1.16 (-2.21 to -0.10)	**0.031**	-0.83 (-1.87 to 0.21)	0.116
Master degree/above	-5.76 (-9.71 to -1.80)	**0.004**	-1.78 (-5.73 to 2.17)	0.377
**Medical institution**				
Tertiary hospital	Ref		Ref	
Secondary hospital	1.82 (0.71 to 2.93)	**0.001**	0.80 (-0.29 to 1.90)	0.149
Community hospital	0.89 (-0.22 to 2.00)	0.117	0.15 (-0.99 to 1.28)	0.801

Ref, Reference category; CI, Confidence Interval, ICU, Intensive Care Unit, β, Unstandardized Coefficient. Bold values indicate statistical significance (*p* < 0.05).

## Discussion

Population aging is a significant challenge faced by many countries worldwide. According to data from the World Health Organization (WHO), the proportion of the global population aged 60 and above is projected to nearly double from 12% in 2015 to 22% by 2050 (10). Currently, the older adult population constitutes about one-tenth of China’s total population, and it is rapidly increasing at an annual rate of 3.8% (11). Projections suggest that the population of Chinese individuals aged 80 and above will soar from 2.7 million in 2020 to over 100 million by 2050, indicating a potential rise in the incidence and severity of frailty syndrome. FS is a common geriatric condition characterized by a decline in physical, psychological, and social functioning, leading to increased risks of disability, reduced quality of life, hospitalization, and death (12, 13). Therefore, effective prevention, screening, and treatment strategies for FS are crucial components of care for older adults (14).

Nurses, as integral members of multidisciplinary teams, play a pivotal role in the management of individuals with frailty syndrome. They possess not only specialized clinical skills and professional knowledge but also the capacity to address the holistic physical, psychological, and social needs of older adults. To comprehensively understand the knowledge, attitudes, and practices (KAP) of nurses regarding frailty syndrome in Fujian Province, this study employed a structured questionnaire, with 598 nurses responding. The majority of participants were female, aged between 30 and 39 years, with college-level education backgrounds. Their work settings were diverse, encompassing internal medicine, surgery, geriatrics, emergency, and intensive care units.

The findings of this study demonstrate an imbalanced knowledge profile among nurses. While most participants retained a solid theoretical grasp of the definition of frailty syndrome, their clinical competency in identifying key risk factors was insufficient, with correct identification rates below 50%. According to the British Geriatrics Society (BGS), CGA is regarded as the gold standard for identifying frailty in older adults, facilitating the development of individualized care plans and reducing hospitalization risks (10). However, this study highlights significant gaps in awareness of frailty assessment criteria and risk factors, consistent with earlier literature suggesting insufficient frailty-specific training in nursing curricula. Additionally, the presence of misconceptions in patient education, cognitive training, and fall prevention further underscores the necessity for targeted education and continuing professional development in frailty prevention and care for older adults.

Nurses generally exhibited a positive attitude toward frailty syndrome, with most acknowledging the importance of frailty prevention and expressing willingness to participate in patient and community education. Notably, 95.4% of respondents agreed that nurses should conduct dynamic frailty assessments for hospitalized patients. However, a quarter of nurses disagreed or strongly disagreed with the view that poor nursing care constitutes a critical risk factor for frailty syndrome. This suggests that while nurses recognize their role in frailty management, some may underestimate the direct impact of nursing care quality on frailty progression. Yet, recent evidence has demonstrated the effectiveness of nurse-led interventions in improving frailty outcomes in older adults (15, 16). Such interventions not only demonstrate scalability and cost-effectiveness but also promote health-related behavioral changes and reduce hospitalization risks (17, 18). Therefore, future geriatric care training should emphasize the critical role of nursing interventions in preventing and managing frailty.

A particularly important finding of this study is the discrepancy between knowledge and attitudes versus actual practice. While the majority of nurses possessed a positive attitude toward frailty syndrome and recognized its importance, there were clear deficiencies in clinical practices such as frailty health education and sarcopenia counseling. Nearly half of the nurses reported rarely or never engaging in health education related to frailty or sarcopenia, whereas education on exercise, healthy lifestyle, and mental health achieved relatively higher implementation rates. This divergence may be attributed to the current lack of structured frailty-specific training programs for nurses. Over the past two decades, sarcopenia has often been the primary focus of basic scientific research, while the concept of frailty has gained greater traction in clinical settings (13, 14, 19, 20). However, the absence of a unified conceptual framework and clear clinical guidelines for managing both conditions likely contributes to these practice inconsistencies.

In the univariate analysis, we observed that nurses with advanced age, longer tenure, and higher educational backgrounds exhibited lower practice scores. However, a crucial finding from our multivariate linear regression model is that these demographic variables lost their statistical significance after adjusting for attitude. This contradicts the assumption that senior or highly educated nurses are inherently less engaged in frailty care due to burnout or administrative burdens. Instead, it suggests that the observed performance gaps are likely mediated by attitude. Essentially, when a nurse possesses a positive attitude, their age, education, or rank does not negatively impede their clinical practice. This indicates that “attitude” acts as an equalizer in the workforce.

Interestingly, nurses working in secondary hospitals displayed higher practice behaviors compared to those in tertiary hospitals. This may reflect the differing care priorities between hospital tiers. Secondary hospitals often manage a higher proportion of older patients with chronic diseases, where frailty assessment and management are integral to long-term care. Conversely, tertiary hospitals focus predominantly on acute and critical care, which may inadvertently deprioritize comprehensive geriatric interventions such as frailty screening and education. This underscores the need for balanced frailty management protocols across all hospital tiers.

Another notable observation was the negative association between higher educational attainment and practice behaviors. Nurses holding college and postgraduate degrees exhibited lower practice scores than those with vocational training. This may be explained by the tendency of higher-educated nurses to assume administrative, academic, or research roles, resulting in reduced direct patient care responsibilities. In contrast, vocationally trained nurses often remain engaged in bedside care, providing them with more opportunities to identify frailty-related issues and implement relevant interventions.

Furthermore, the multiple linear regression analysis clarified the mechanism driving practice behaviors. While knowledge showed a positive correlation in univariate analysis, it was not a significant independent predictor in the multivariate model (β = 0.05, *p* = 0.359). In contrast, attitude emerged as the sole robust predictor (β = 0.67, *p* < 0.001). This discrepancy highlights a distinct “Knowledge-Practice Gap”: possessing theoretical knowledge about frailty is a necessary foundation but is insufficient on its own to drive behavioral change. Our findings align with the Theory of Planned Behavior, suggesting that intrinsic motivation (attitude) is the primary engine that converts latent competence into actual bedside practice.

### Perspectives for clinical practice in a public health context

Interpreting these results through the lens of the Quadruple Aim (5) (improving population health, reducing costs, enhancing patient experience, and improving provider work-life) suggests that the current nursing model for frailty management requires urgent “renovation.”

First, the finding that attitude overrides demographic factors has profound implications for workforce management. It suggests that clinical pathways should not simply target “younger” or “less educated” nurses for execution. Instead, interventions must focus on fostering a proactive professional culture. Since frailty care often involves preventive, non-urgent interventions (e.g., health education) that are easily deprioritized, hospital administrators must establish multidisciplinary clinical networks that recognize and reward these “invisible” safety measures.

Second, the “Knowledge-Practice Gap” necessitates a shift in training strategies. Traditional didactic lectures may increase knowledge scores but fail to change behavior. To ensure Patient Safety, training programs must incorporate motivational strategies and case-based simulations that enhance nurses’ self-efficacy and professional value, thereby boosting the “Attitude” factor which our study proves is the decisive driver of practice.

### Limitations

This study has several limitations. First, the reliance on self-reported data introduces the possibility of recall and social desirability bias. Second, the sample was limited to nurses from Fujian Province, which may affect the generalizability of the results to other regions with different healthcare structures or educational backgrounds. Third, as the survey was voluntary, selection bias cannot be entirely ruled out; nurses with a pre-existing interest in frailty may have been more likely to participate. Due to the anonymous distribution method via WeChat, demographic data for non-responders could not be collected for comparison. However, the gender (97.5% female) and age distribution of our sample generally align with the known workforce demographics of registered nurses in Fujian Province, suggesting the sample remains reasonably representative. Fourth, we acknowledge that the phrasing of certain attitude items might have been leading, potentially eliciting socially desirable responses. While this may explain the high attitude scores, the strong correlation between these scores and practice behaviors in our linear model confirms their predictive validity despite this limitation. Fifth, this study primarily focused on hospital-based nurses, leaving the KAP status of those in community healthcare settings or nursing homes unexplored. Given the pivotal role of community-based geriatric care in managing frailty, future research should extend to these environments. Finally, a methodological limitation concerns the validation of the questionnaire. While the instrument underwent a rigorous content validity review by a multidisciplinary panel of experts and was grounded in established clinical guidelines, we did not perform *post hoc* statistical analyses to evaluate its psychometric properties, specifically internal consistency (e.g., Cronbach’s alpha) or construct validity. Consequently, the statistical stability and homogeneity of the scale items remain unverified. Therefore, the results should be interpreted with a degree of caution, and future studies are encouraged to utilize or develop psychometrically validated instruments to confirm these findings.

## Conclusion

In conclusion, this study highlights both the strengths and deficiencies in the knowledge, attitudes, and practices of nurses regarding frailty syndrome in Fujian Province. The findings emphasize the need for comprehensive, standardized training programs that prioritize early identification and preventive strategies, particularly for pre-frail older adults. Addressing these gaps through targeted educational interventions and organizational policy support will not only enhance the quality of care but also facilitate early interventions to delay the progression of frailty in the rapidly aging population.

## Data Availability

The raw data supporting the conclusions of this article will be made available by the authors, without undue reservation.

## Supplementary material

Supplementary Material 1 Completed STROBE statement checklist.

Supplementary Material 2 Questionnaire design, scoring criteria, and item explanations.
